# ‘I thought if I marry the prophet I would not die’: The significance of religious affiliation on marriage, HIV testing, and reproductive health practices among young married women in Zimbabwe

**DOI:** 10.1080/17290376.2016.1245627

**Published:** 2016-10-20

**Authors:** Denise Dion Hallfors, Bonita J. Iritani, Lei Zhang, Shane Hartman, Winnie K. Luseno, Elias Mpofu, Simbarashe Rusakaniko

**Affiliations:** ^a^ PhD Senior Research Scientist, is affiliated to Pacific Institute for Research and Evaluation, 1516 E. Franklin St., Suite 200, Chapel Hill, NC 27514, USA; ^b^ MS, MA Associate Research Scientist, is affiliated to Pacific Institute for Research and Evaluation, 1516 E. Franklin St., Suite 200, Chapel Hill, NC 27514, USA; ^c^ PhD Associate Research Scientist, is affiliated to Pacific Institute for Research and Evaluation, 1516 E. Franklin St., Suite 200, Chapel Hill, NC 27514, USA; ^d^ BA Research Associate, is affiliated to Pacific Institute for Research and Evaluation, 1516 E. Franklin St., Suite 200, Chapel Hill, NC 27514 USA; ^e^ PhD Research Scientist, is affiliated to Pacific Institute for Research and Evaluation, 1516 E. Franklin St., Suite 200, Chapel Hill, NC 27514, USA; ^f^ PhD, DEd Professor of Rehabilitation Counseling, is affiliated to Department of Rehabilitation Counseling, Faculty of Health Sciences, University of Sydney, Australia; ^g^ PhD Professor and Chairman, is affiliated to Department of Community Medicine, University of Zimbabwe, PO Box A 178, Avondale, Harare, Zimbabwe; ^h^ PhD, DEd Professor of Rehabilitation Counseling, is affiliated to Department of Research and Innovation, Central University of Technology, Bloemfontein, South Africa

**Keywords:** Zimbabwe, marriage and religion, women’s reproductive health behaviors, HIV testing, qualitative research, Zimbabwe, le mariage et la religion, les comportements de santé reproductive des femmes, dépistage du VIH, la recherche qualitative

## Abstract

This study examines the association between religious affiliation and reasons for marriage, perceived church attitudes, and reproductive health-seeking behaviors, including HIV testing, among young women in eastern rural Zimbabwe. The sample comprised women (*N* = 35) who had married by 2012 while participating in a larger randomized controlled trial (RCT) to test the effects of school support on HIV-related risk. The RCT sample was identified in 2007 as all female sixth graders in 25 rural eastern Zimbabwe primary schools whose parents, one or both, had died (*N* = 328). In our previous RCT analyses, we found that participants who affiliated with an Apostolic church were more than four times more likely to marry than those from non-Apostolic churches and that control group participants were twice as likely to marry as those in the intervention group. Other studies had found that marriage greatly increased the odds of HIV infection among adolescent women. Given the link between Apostolic affiliation and marriage, we conducted semi-structured interviews to explore type of marriage, reasons for marrying, church affiliation and attitudes, family planning, HIV testing, schooling, and family life. We were interested in differences, as perceived by our sample of young married women congregants, among Apostolic sects and other denominations in their attitudes about marriage and health-seeking behaviors. We were also interested in the influence of church affiliation on intervention participants’ decision to marry, since they had comprehensive school support and education is highly valued in Zimbabwe, but costly and often out of financial reach. Interviews were conducted from October 2012 through November 2013; data were analyzed using a general inductive approach. We found that pressure or perceived deception for coitus or marriage was reported only by intervention participants affiliated with Apostolic denominations. Other reasons for marriage were similar between Apostolic and non-Apostolic adherents, as well as intervention and control conditions. All participants believed HIV testing was important, but while all non-Apostolic denominations encouraged HIV testing and clinic/hospital care, there was considerable heterogeneity in attitudes among Apostolics, with ultraconservative denominations most likely to proscribe non-religious health care. We conclude that some, but not all, Apostolic-affiliated women are afforded discretion in their health-seeking behaviors. Since HIV screening and treatment depend on access to clinic/hospital care, continued public health efforts to engage Apostolic leaders is needed, along with monitoring of progress in access and outcomes.

## Résumé

Cette étude porte sur l'association entre l'appartenance religieuse, les raisons de se marier, les attitudes perçues de l'église et les comportements favorables à la santé en matière de reproduction, y compris le dépistage du VIH, parmi des jeunes femmes à l'Est du Zimbabwe rural. L'échantillon comprenait des femmes (*N* = 35) qui s’étaient mariées en 2012 et qui participaient en même temps à un essai contrôlé randomisé (RCT) plus grand, destiné à tester les effets du soutien scolaire sur le risque lié au VIH. L'échantillon RCT en 2007 étaient constitué de tous les élèves féminins de 6^e^ année dans 25 écoles primaires rurales de l'Est du Zimbabwe dont les parents, un ou les deux, étaient morts (*N* = 328). Dans nos analyses RCT précédentes, nous avons constaté que les participants qui appartenaient à une église apostolique étaient quatre fois plus susceptibles de se marier que ceux des églises non-apostoliques, et que les participants du groupe témoin étaient deux fois plus susceptibles de se marier que ceux du groupe intervention. D'autres études ont montré que le mariage augmentait considérablement les chances d'infection au VIH chez les adolescentes. Étant donné le lien entre le fait d’appartenir à un groupe religieux apostolique et le mariage, nous avons mené des entrevues semi-structurées pour explorer ce qu’il en est du type de mariage, des raisons de se marier, de l'appartenance et des attitudes religieuses, de la planification familiale, du dépistage du VIH, de la scolarité et de la vie de famille. Nous étions intéressés par les différences, telles qu'elles sont perçues par notre échantillon de jeunes fidèles mariées, au sein des sectes apostoliques et d'autres confessions, dans leurs attitudes sur le mariage et les comportements favorables à la santé. Nous étions aussi intéressés par l'influence de l'appartenance religieuse sur du groupe intervention dans la décision de se marier, car la prise en charge complète de l'école et de l'éducation est très appréciée au Zimbabwe, mais cher et souvent hors de portée financière. Les entrevues ont été réalisées d’octobre 2012 à novembre 2013. Les données ont été analysées à l'aide d'une approche inductive générale. Nous avons constaté que le recours à des moyens de pression ou à ce qui était perçu comme de la tromperie pour obtenir une relation sexuelle ou le mariage n’a été signalé que par les participants d'intervention appartenant aux groupes religieux apostoliques. D'autres raisons pour le mariage étaient similaires entre fidèles apostoliques et non-apostoliques, ainsi que l'intervention et les conditions de contrôle. Tous les participants ont estimé que le dépistage du VIH était important, mais alors que tous les groupes religieux non-apostoliques encouragent le dépistage du VIH et les soins à la clinique ou à l’hôpital, il y a une grande hétérogénéité dans les attitudes des Apostoliques ; les groupes religieux ultraconservateurs étant les plus susceptibles de proscrire les soins de santé non-religieux. Nous concluons que certaines femmes (mais pas toutes) appartenant à des groupes religieux apostoliques, disposent d’une certaine latitude dans leurs comportements de santé. Puisque que le dépistage et le traitement du VIH dépendent de l'accès à des soins en clinique ou à l'hôpital, la poursuite des efforts de santé publique pour impliquer les dirigeants apostoliques est nécessaire, ainsi que le suivi des progrès dans l'accès et les résultats.

## 1. Introduction

Religion^1^ is a powerful influence in the lives of southern Africans, affecting all aspects of daily life and health, particularly for rural women (Agadjanian, 2005; Chitando, 2007). Rural churches are a center for informal social interaction, as well as formal teaching and regulation, all of which shape attitudes about health-seeking behaviors (Agadjanian & Menjivar, 2008; Collaborating Center for Operational Research and Evaluation [CCORE], 2011; Mpofu et al., 2011). Although the influence of religious organizations has been recognized as important in the HIV/AIDS epidemic (e.g. Dilger, Burchardt, & van Dijk, 2010; Klaits, 2005; Olivier et al., 2015), few studies have examined how religious affiliation might affect early marriage and reproductive health behavior. This paper examines the association between religious affiliation and reasons for marriage, perceived church attitudes, and reproductive health-seeking behavior, including HIV testing, among young women in rural eastern Zimbabwe.

Prior to the arrival of European colonial missionaries, the primary religious belief system in Zimbabwe was Shona. Catholic and Protestant missionaries were highly successful in setting up new church congregations, schools, and hospitals during the colonial era and recruited large numbers of members (Gregson, Zhuwau, Anderson, & Chandiwana, 1999; Patterson, 2011). Christianity was readily accepted, in part because of its compatibility with Shona beliefs, although these beliefs held much greater emphasis on the involvement of benevolent and malevolent spirits in everyday affairs, and included the concept of ancestral spirits interceding to God for living people (Chitando, 2007; Moyo, 1988). Starting in the 1940s, exclusion of non-European Zimbabwean men from senior mission church leadership positions and marginalization of their religious heritage contributed to emergence of African-initiated ‘Spirit-led’ Christian churches in Zimbabwe (Daneel, 1987; Patterson, 2011). One of the largest African-initiated churches, and among the largest denominations in Zimbabwe, is the African Apostolic Church of Johane Marange (Anderson, 2001; Nenge, 2013). Marange renounced ritual practices and witchcraft, exhorted the keeping of biblical Old Testament laws, and emphasized the workings of the Holy Spirit in healing and leading the people (Anderson, 2001).

While the Johane Marange Church is prominent, there are many other Apostolic sects in Zimbabwe, often formed under the leadership of a charismatic healer and prophet (Anderson, 2001; Daneel, 1987). The most recent population-based Zimbabwe Demographic and Health Survey identified Apostolics as the largest single religious denomination in the country with 38% of women and 28% of men ages 15–49 (Zimbabwe Central Statistical Office & Macro International Inc., 2011). Other religious affiliations reported among Zimbabwean women and men, respectively, were Pentecostal (21%, 15%), Protestant (17%, 14%), Roman Catholic (8%, 10%), Other Christian (8%, 8%), no religion (6%, 22%), traditional (1%, 4%), and Muslim (1%, 1%).

We conducted a randomized controlled trial (RCT) of orphaned girls in rural eastern Zimbabwe to test the impact of school support as a structural approach to prevent HIV risk factors, including child marriage (Hallfors et al., 2011, 2015). Orphans in sub-Saharan Africa are more vulnerable than non-orphans to delayed progression in school and dropout (Mishra & United States Agency for International Development, 2005; Pufall et al., 2014), early marriages, and HIV infection (Birdthistle et al., 2009; Gregson et al., 2005; Luseno, Zhang, Rusakaniko, Cho, & Hallfors, 2015). According to the most recent population-based data (Zimbabwe National Statistics Agency [ZIMSTAT] & ICF International, 2012), HIV prevalence is 17.7% among women aged 15–49 and 12.3% among men aged 15–54. Due in large part to Zimbabwe’s severe and protracted HIV epidemic, 21% of children under 18 years and 41% of adolescents aged 15–17 years are orphaned, with either one or both parents dead. Thus, young orphaned women represent a large and vulnerable group in Zimbabwe, important to public health.

During our study, we observed the widespread influence of Apostolic sects and found that affiliation greatly increased the likelihood of dropping out of school by participants to marry men often much older than themselves (Hallfors et al., 2013). Thus, after the last wave of survey data collection (2012), we invited those who reported having ever married to participate in a qualitative interview. Very few studies are available in the published peer-reviewed literature about possible church influences on women’s health in southern Africa, but available reports show associations between Apostolic affiliation and public health practices and outcomes. Because most Apostolic sects espouse faith healing over medical care, members have lower rates of skilled birth attendance, antenatal care visits, and lower rates of childhood immunizations compared to other denominations (Ha, Salama, Gwavuya, & Kanjala, 2012). Apostolic churches are more likely to shun modern medical procedures, medicines, and modern family planning methods and are less likely to support HIV prevention, care, and treatment compared to Catholic or Protestant denominations (Gregson et al., 1999; Mpofu et al., 2011; Nhamo et al., 2011). Apostolic affiliation has been associated with increased measles cases (Pomerai, Mudyiradima, & Gombe, 2012), perinatal mortality (Emmanuel et al., 2011; Munjanja, 2007) and maternal mortality (Munjanja, 2007).

Much of the research about Apostolic practices, however, has not distinguished between different sects, despite heterogeneity in church teaching, exhortation, and control of member behavior (CCORE, 2011; Nhamo et al., 2011). For example, some Apostolic sects preach against modern medical care and only endorse faith healing, while others endorse both (Nhamo et al., 2011). ‘Ultraconservative’ sects (CCORE, 2011) may account for poor maternal and child health outcomes associated with Apostolic membership.

Identified ultraconservative sects include the Johane Marange and Johane Masowe Apostolic Sects (CCORE, 2011). The Johane Marange sect, which originated in Manicaland Province, has an estimated 1.2 million members in Zimbabwe (Sibanda, 2011). It is an insular group that practices polygamy and child marriage (CCORE, 2011; Mavungandize, 2008), including, in some cases, forcing girls to marry elderly men based on prophecy (Nenge, 2013). The Marange church curtails education for women (Nenge, 2013), and it is common for young adolescent girls’ schooling to be stopped during the religious festival in July during which they may be married (Child Rights Information Network [CRIN], 2011). The church also endorses male patriarchy in marriage and restrictive practices toward women’s activities and decision-making, including the use of modern medical services (CCORE, 2011; Nenge, 2013). Pregnant women in the ultraconservative Apostolic sects are less likely than others to use professionally assisted antenatal and delivery care and instead are attended by untrained midwives (CCORE, 2011). The two adolescent participants in our own study who died due to complications from giving birth were both married to men from the Johane Marange sect (Hallfors et al., 2015).

The religious teaching, doctrine, and regulations of the ultraconservative groups are thought to undermine modern health-care seeking through sanctions which include confession and shaming (CCORE, 2011). On the other hand, semi-conservative Apostolic groups are reported to have ambiguous teachings and church doctrine related to use of modern health-care services, and rather than imposing sanctions, they encourage members to seek spiritual counsel and faith healing before using modern health-care services (CCORE, 2011). Examples of semi-conservative Apostolic groups include Africa Apostolic Church, Zviratidzo Zvemapostori, Mughodi Apostolic Church, Jekenisheni, and Zion Apostolic Church.

It is important to study the influence that different church denominations may have on young women’s lives, including child marriage and health-seeking behaviors related to HIV prevention. Other studies have used geographic data (Gregson et al., 1999), national data sets (Ha et al., 2012), and focus groups in an attempt to examine religious influences and health outcomes in the general population (Nhamo et al., 2011). Confidential individual interviews of young women in a high HIV-prevalence area are lacking in the literature, yet needed to understand structural and social factors affecting their health choices.

In the present paper, we present data from interviews with young women in Zimbabwe and examine the influence of religious affiliation on constructs of interest. In our previous report (Hallfors et al., 2013), we found that religion was highly important to the majority of participants in our RCT study. This paper uses qualitative methods and general inductive analyses to examine the association between religious affiliation and reasons for marriage, perceived church attitudes, and reproductive health-seeking behaviors, including HIV testing, of participants who married during the course of the RCT. The study is unique in probing denominational distinctions in young women’s perceptions about marriage and reproductive health practices among a hard-to-reach population.

## 2. Methods

### 2.1. Participants

Participants were from a rural sample of girls who participated in a RCT in Manicaland province, Zimbabwe. Detailed information on purpose, design, and data collection procedures is presented elsewhere (Hallfors et al., 2011, 2015). Briefly, the RCT was designed to test whether supporting orphan adolescent girls to stay in school would prevent HIV risk factors and infection. Participants comprised all orphan girls (*N* = 328) in the sixth grade in 2007 from 25 primary schools in a rural Shona-speaking province of Zimbabwe. Schools were randomized to either an intervention group (receiving school fees, uniforms, school supplies, and a school-based helper through the end of high school) or a no-treatment control group. In January 2011, the control group was offered school fees as a delayed partial intervention (Hallfors et al., 2015).

Eligible participants for the present marriage study were those from both conditions who had completed the final RCT survey in 2012, had indicated in the survey that they had ever been married, and who agreed to be interviewed. The semi-structured marriage interviews were conducted from October 2012 through November 2013. Of 44 eligible participants, 35 participated, of whom 13 were from the intervention group and 22 from the control group. Of those who did not participate, eight could not be located for interview and one refused.

### 2.2. Procedures for qualitative interviews

Interviews were conducted in the respondent’s home, or a place of their choice, by one of two trained Zimbabwean women, both with master’s degrees in psychology. Interviews were conducted in the local language of Shona and took about 60 minutes. The interviewer used a semi-structured guide with probes to ensure that individual questions were answered. A trained female Zimbabwean with a business administration undergraduate degree transcribed the interview in shorthand. Interview questions explored type of marriage (monogamous or polygamous; traditional or registered marriage), reasons for marrying, church affiliation and attitudes, family planning, HIV testing, schooling, and family life. Questions were developed based on our RCT findings of much higher rates of marriage at earlier ages among young women who were either Apostolic or who married Apostolic men; focus group interviews conducted with men and with women from one Apostolic sect (Mpofu et al., 2011); anecdotal information from health and school officials; and published studies from the literature. Interviewers were trained to be non-judgmental in their attitudes and questions, respecting the views and values of the participants, and were observed by a native Zimbabwean Co-investigator in pilot interviews.

The shorthand notes were transcribed in Shona and translated into English, and the interviewer reviewed and made any needed corrections to the transcript. After reviewing the first two interviews, the lead investigators met with the interview team, provided feedback and further training, and fine-tuned the interview procedures. The investigators reviewed all transcripts asking for any clarification as needed and monitoring for participant safety.

The in-country lead investigator provided training on protocols and ethical issues specific to the study to the two interviewers and transcriber. All participants provided signed informed consent prior to interviews. The institutional review boards of (the Pacific Institute for Research and Evaluation) and the Medical Research Council of Zimbabwe approved all study procedures.

Religion type was determined by the participant’s answer to her current denominational affiliation. Researchers also asked whether she had belonged to another denomination before marriage. Based on classifications from the literature (Patterson, 2011) and Zimbabwean researchers knowledgeable in local religions, we categorized denominations as follows: (1) Apostolic sects: Africa Apostolic Church, Jekenisheni, Johane Marange Apostolic Church, Johane Masowe, Johane Masowe Echishanu, Kuera Kwemasimba Emapositori, Pentecost, Zion, Zion Christian Church, Zviratidzo Zvevapositori, and Mugodhi; and (2) non-Apostolic churches: Anglican, United Methodist, Roman Catholic, and three Pentecostal sects: Apostolic Faith Mission, Zimbabwe Assembly of God (ZAOGA), and Christian Faith Ministries.

### 2.3. Analyses

Using general inductive analyses, we read and reread transcripts and identified and coded common topics related to reproductive and HIV health behaviors and attitudes that emerged from the data (Ulin, Robinson, & Tolley, 2005). Themes were developed from the coded text and grouped accordingly. Each thematic category could contain sub-codes that were developed during the reading process. Using the final list of codes, all transcripts were read and coded independently by two trained investigators. Inter-rater reliability for qualitative coding across each theme ranged from 80% to 100%. Where discrepancies occurred, the reviewers discussed, clarified codes as needed, and came to final consensus. Quotes from transcripts are presented verbatim to exemplify themes. Condition (intervention or control) of participants is noted in themes related to reason for marriage only, since we were interested in the circumstances under which intervention participants would choose marriage over school continuation when given full support to stay in school, and how these reasons might be related to denominational affiliation. For all analyses, only descriptive statistics are reported, with no significance testing.

## 3. Results and analysis

We found that all participants were in a ‘traditional’ Zimbabwean marriage. This meant that their marriages were not registered with the government, but required *lobola*, or a bride-price paid to the woman’s family. Two of the women said that their marriages had been ‘blessed’ (recognized publicly, with a blessing from congregational leaders), but none were licensed with monogamous vows in the presence of a marriage officer as specified in the Zimbabwe Marriage Act (Chirawu, 2006).

Age discrepancy between husband and wives was large in almost all marriages in our sample, but larger among Apostolics compared to non-Apostolics (Table 1). For participants, the median age at marriage for Apostolics was 16 years compared to 17 years for non-Apostolics, and median years of schooling were 7.5 vs. 8.0, respectively. For husbands, the median age at marriage for Apostolic husbands was 24 years compared to 21 years for non-Apostolics and median years of schooling were 10.0 vs. 11.1

**Table 1.** Religious affiliation and spousal demographics by Church type.

	Apostolic (*n* = 22)	Non-Apostolic (*n* = 13)
Participant		
Mean age at marriage (range)	16.6 (13.0–21.0)	17.2 (15.0–23.0)
Median age at marriage	16.0	17.0
Median years of schooling (range)	7.5 (6.0–10.0)	8.0 (6.0–9.0)
Husband		
Mean age at marriage (range)	25.0 (17.0–48.0)	22.6 (18.0–35.0)
Median age at marriage	24.0	21.0
Median years of schooling (range)	10.0 (3.0–13.0)	11.1 (10.0–13.5)
Participant’s Original Affiliation^a^		
From different church type	7 (32%)	1 (8%)
Same type, different denomination	6 (27%)	7 (54%)
Same type, same denomination	9 (41%)	5 (38%)

^a^Note that one non-Apostolic women had married an Apostolic man but then left him and returned to her original non-Apostolic denomination. Thus, she is categorized here as non-Apostolic.

### 3.1. Church affiliation

Of the 35 interviewed participants, 22 were currently affiliated with Apostolic sects and 13 were affiliated with non-Apostolic churches (Table 1). With marriage, wives joined their husband’s family church, regardless of their affiliation prior to marriage as is customary practice in Zimbabwe. Among Apostolic wives, seven were originally from non-Apostolic churches, six were from other Apostolic sects, and nine married men from their own sect. Among wives from non-Apostolic churches, only one originally came from an Apostolic Sect, seven came from a different denomination, and five married men from their own denomination. One had married an Apostolic man but then returned to her non-Apostolic church after their marriage broke up.

Wives who left one church for another often noted important differences in practices that had significant impacts on their lives. This young woman, a former member of a non-Apostolic Pentecostal church, married a man in a conservative Apostolic church that encouraged polygamy and faith healing:

It is different from my first church ZAOGA. ZAOGA encourages white wedding [licensed monogamous marriage] and does not encourage polygamist. Johane Masowe does not believe in going to clinics. When you are sick you will be given holy water and other things such as mazoe and milk depending on what the Holy Spirit will have revealed.

This young woman abided by the new church regulations and did not go to a clinic or hospital for her pregnancy or delivery and had never been HIV-tested. Nevertheless, she told the interviewer she was happy in her marriage and was her husband’s only wife.

Another young woman, formerly a member of an Anglican church, had married a man from Johane Marange Apostolic church and was the latest of nine wives. She found that the new church’s expectations about *lobola* were different from her own family church, causing conflict and distress. Although the white robe specific to the Marange church would have sufficed as payment to the bride’s relatives among sect members, the young woman’s non-Apostolic brother had civil standing to go to the magistrate and require a larger settlement.

They do not encourage the type of lobola required in other churches or in the community, the husband is supposed to buy his in laws the church uniform only. For me it was very difficult because my brother is not in Johane Marange Apostolic church and he demanded the actual lobola. My husband refused to pay it and the case was reported to the police. He later agreed to give him US$250 and he said he will need more time for him to pay the lobola.

### 3.2. Reasons for marriage

There were few differences between Apostolic and non-Apostolic-affiliated participants in most categories of ‘reasons for marriage’. Reasons included: pregnancy; family consequences related to suspected loss of virginity; no alternatives; pressure or perceived deception for coitus or marriage; and personal choice. All church denominations forbade sexual intercourse before marriage and many required the couple to marry if found out. Most young women, regardless of specific religious affiliation, married because of pregnancy or family consequences for staying out too late into the night with their boyfriends. When asked, ‘What made you decide to get married?’ approximately one-third of participants affiliated with each church type (Apostolic and non-Apostolic) responded that they had become pregnant. Three of the 11 in this group were from the intervention condition. Even if not pregnant, about 20% more from each church type reported that they were immediately evicted from their home by a family member who believed their virginity was compromised.

I went to see my boyfriend and we were together until it was very dark. When I got home my mother chased me away. She told me to go back where I was so I went to my boyfriend … He agreed to marry me and he took me to his family home. (Apostolic woman)

Since one or both of the participants’ parents were dead, siblings or other relatives with whom they lived might be the ones to evict them. ‘My brother saw me with my boyfriend and he told me not to come home again. My boyfriend agreed to stay with me’ (Non-Apostolic woman). Even if not chased away by a family member, many young women believed that sexual/romantic behavior compromised their place in the family.

I left school in form one (high school) and fell in love with my boyfriend and we stayed out late and I was afraid to go home. I knew if I had gone, [I] was going to be chased away. (Apostolic woman)

Only four young women, two affiliated with Apostolic (9%) and two with non-Apostolic denominations (15%), mentioned personal choice as a reason for marriage, while 23% from each of the two church types said that poverty, poor grades, or ill treatment at home led them to marriage (no alternatives). Three of the eight young women in this latter group were from the intervention condition. This Roman Catholic participant described an intolerable situation living with her aunt as the impetus to marry.

My aunt was taking care of me and my brother. She would give good food to her children only. My brother and I would eat different food from what they will have ate. If you make a mistake she would beat you up and says if you want you can go to the graveyard to stay with your mother. I had no peace at that house until I decided to tell my boyfriend the situation I was in. He agreed to take care of me and I went to stay with him.

An Apostolic participant, a member of the intervention group, decided that even with a scholarship, there was no reason to stay in school.

I was not bright in school so I decided that going to school was just wasting resources. I never passed any subject. I dropped out of school and decided to marry.

Similarly, a second Apostolic intervention participant said she had dropped out of school and so decided to marry. On the other hand, four control group participants (three Apostolic, one non-Apostolic) gave reasons for marriage similar to this Africa Apostolic young woman, ‘There was no one who was paying for my school fees so I decided to get married’.

Five participants said that they had been pressured or deceived into coitus or marriage. All five were affiliated with Apostolic sects; two of these had left non-Apostolic denominations when they married. All five were from the intervention condition. The first, a Zion Apostolic, presents an example of perceived deception.

My husband … pretended to prophecy that one member from our family was going to die and since I was still young I thought if I marry the prophet I would not die.

Two other participants entered a polygamous marriage in the Johane Marange sect. One entered a marriage arranged by her father and husband.

My sister who was married to my husband died. An agreement for a replacement was signed for between my father and my husband … I only came to know about the replacement agreement the day my husband came to take me.

The second young woman was lured into marriage by a cousin, one of eight wives, in an attempt to make the most recent wife jealous. The interviewer recorded her observations after visiting the young woman at her home for the interview. She noted that seven of the wives were living together with their children in one large room, while the husband had his own room. All worked in a plot of ground to raise food to sell. The husband allowed them one day/week to work in other peoples’ fields for pocket money or for their children’s school fees. The participant said she was thinking of going to town to find work as a housemaid but she is not sure if the husband will allow this. The interviewer observed that the young woman and her baby were not getting enough to eat.

Two of the women who felt they had been deceived had left their husbands by the time of the interview. One Zion woman explained the breakup as follows:

I never enjoyed my marriage. My husband was not taking care of me and used to demand that I have to do piece works so that I buy him cigarettes but I did not have the money for that. The little money that I will have worked for was enough to feed the family.

A second woman, who believed her husband’s family had colluded so that he could have sex with her, returned to her United Methodist congregation after leaving her Mughodi Apostolic husband.

I had a less happy marriage than others. My husband started going out with other girls in school and he would be seen by my family members with other girls.

Interestingly, a sixth participant, from the control condition and a Johane Marange congregation, eloped rather than enter an arranged marriage, and so was classified ‘personal choice’ as reason for marriage. Her husband was a Mughodi Apostolic.

A sister who was married died. After a year, Father and brother were arranging that I go and stay with the late sister’s husband. I discussed the issue with my boyfriend and we then agreed that I elope. I wanted to stay with a man of my choice.

### 3.3. Church attitudes toward polygamy and child marriage

We asked participants about their church’s attitudes about polygamy and child marriage (described in our interview guide as men over 21 years marrying girls 16 years and younger). Most of the young women were very opposed to both practices. Apostolic-affiliated young women, and particularly those in ultraconservative Apostolic sects, were more likely to report church support for these practices, but attitudes and sanctions varied across denominations. As can be seen in quotes below, some Apostolic denominations do not allow child marriage but may accept the union if the girl agrees and is not being coerced. Non-apostolic denominations typically object to both child marriage and polygamy, but some may limit sanctions to leaders, or may defer to the girl’s parents.

The church does not have problems with that [child marriage]. It is common in our church. (Johane Masowe Apostolic)

It is not accepted although some people do it. If the two love each other there is no problem but if the man is forcing the girl the church elders will try him and suspend him from the church. (Jekenisheni Apostolic)

Usually it [polygamy] only affects leaders as you will be asked to step down but if you do not have any position they will just caution you on the matter … It [child marriage] is not encouraged but no action is taken to those who will have done it. (Apostolic Faith Mission, a non-Apostolic Pentecostal denomination)

It is not acceptable but it is up to the parents of the girl to make a decision. If they accept the lobola then they have agreed to marry their daughter at that age. (Anglican woman)

### 3.4. Church attitudes toward reproductive health care

None of the non-Apostolic-affiliated participants said that their churches forbade them to attend clinics for health care and many said that their church actively encouraged practices such as HIV testing, immunization, and family planning. Apostolic-affiliated participants were mixed in their reports of denominational attitudes on reproductive health care. All participants, regardless of religious affiliation, thought it was important to be tested for HIV, but some Apostolic-affiliated participants reported they had never been tested because their denomination and/or husbands did not allow them to do so, with similar directives against immunization and family planning. Among Apostolics, Johane Marange-affiliated participants consistently reported that they were not allowed to go to hospitals or clinics. Two obeyed these directives while one, who had been a lifetime church member, did not. Her husband and her congregation, however, tolerated her independent behavior with few sanctions:

They encourage mothers to go to the church elders with their children so that they will be prayed for. They do not believe in medical treatments. I go to [name removed] clinic with my son for immunization because I have already told them that I will always go to the clinic if I got sick or if my son is sick … I am on family planning method although our church does not allow us to go to clinics or hospital. I use some pills that I get from [name removed] clinic for free. Each time I go to church I will confess that I am taking tablets and I cannot stop them.

Participants affiliated with other Apostolic sects, particularly Johane Masowe, Johane Masowe Echisanu, and Zion, reported different attitudes within the same denomination, with some saying that modern health-seeking behaviors were not allowed, others saying that their church did not talk about it, and still others saying that they were encouraged. The first two quotes are from participants who are affiliated with congregations of the Johane Masowe sect:

The church does not encourage members to go to clinics for testing. The prophets can see that you are HIV positive and they can give you a mixture of honey, milk and cooking oil. If you drink the mixture you will be cured … I was tested. It was required at [name removed] hospital, they test every pregnant woman. My husband has not been tested. He refuses.

It [the church] encourages people to go for HIV testing. I was tested but my husband refused.

The next two quotes are from participants affiliated with two different congregations of the Zion denomination. Leaders in the congregation appear to have opposite attitudes toward HIV testing, family planning, and child immunizations.

It is not allowed to go to clinics and hospitals. Some individuals do that [family planning] secretly so that they will be able to raise their families in a good way … It [HIV testing] is not encouraged in the church, but some members have to decide on their own whether they should be tested or not … Children are not immunized. It is not allowed, however some people do that secretly.

The church encourages members to go to clinics for assistance. It helps people to raise their children well and not have unwanted pregnancy. I am on depo provera. I received the injection for free … The church encourages everyone to be [HIV] tested … It encourages women to go with their children for immunization. My son is receiving immunization injections.

Other Apostolic sects appear to be softening in their views and even encouraging modern health practices as quotes from two participants affiliated with Africa Apostolic Church congregations suggest.

I am on depo provera. It is for free at the clinic. Our church does not encourage family planning but most women now understand the importance of family planning. The church elders are no longer strict on that.

It [HIV testing] is now encouraged but previously they did not allow people to go to hospitals. We have been tested 3 times together and each time I go for my family planning method they start by testing for HIV before attending to you.

About the same proportions of wives in Apostolic (73%) and non-Apostolic (77%) sects said they either approved of modern family planning methods or explicitly stated that they were on birth control. In almost all cases, they said they were on pills or hormonal implants. In only one case did a wife say she and her husband used condoms. Birth control aids were obtained free of charge from local clinics or purchased for a very nominal amount. Most wives had just one child, even if they had been married for several years. Women who were not on birth control had not yet had their first child or they were Apostolic and using other methods, approved by their church. As an example of this latter group:

We are not allowed to go to the clinic or hospital unless the Holy Spirit has said so. If you do not want to be pregnant you will just avoid sex. Since I have gave birth to my child I have never had sex with my husband. (Johane Marange woman in polygamous marriage)

## 4. Discussion

This study examined the associations between religious affiliation and reasons for marriage, perceived church attitudes, and reproductive health-seeking behaviors, including HIV testing, among young women in eastern rural Zimbabwe. Unique to this study, we examined denominational differences among Apostolic sects. We also sought to explore why participants with comprehensive school support dropped out of high school to marry.

Findings suggested few differences in reasons for marriage by church type. Becoming pregnant was a common reason, as was being ‘chased away’ by family members if there was real or suspected sexual indiscretion. In a previous study, we found that the perceived messages most salient to rural Zimbabwe high school girls’ sexual decision-making were biblical teachings, family honor, and social stigma associated with non-marital sex (Mpofu, Hallfors, Mutepfa, & Dune, 2014). This is consistent with present study participants’ reports that marriage was their only option after coitus or pregnancy.

One reason that was reported only by participants who married an Apostolic man, however, was pressure or deception by their husbands, sometimes in coordination with other family members. The relatively larger age difference between Apostolic men and their young wives may exacerbate the potential for abuse and deserves further research. Unexpectedly, we also found that only intervention group participants reported being either pressured or tricked into marriage, sometimes resulting in very difficult marriage and living situations. This finding suggests the need for further research to improve the effectiveness of school support as an HIV prevention strategy, since an earlier seminal Zimbabwe study found that the odds of being infected with HIV are much higher among married, compared to unmarried, adolescent women (Birdthistle et al., 2008).

We also found that finishing high school is not the preferred option for all young women, even with comprehensive support, as reported by two married participants from the intervention group. On the other hand, several control group participants told interviewers that lack of school fees influenced their decision to marry, as they could no longer continue in school. In the RCT study, we found that the school support intervention greatly reduced both school dropout and early marriage among the intervention group compared to controls (Hallfors et al., 2015).

As in previous reports (CCORE, 2011; Daneel, 1987), we found that Apostolic churches were more likely to support polygamy and marriage of young girls to adult men than non-Apostolic churches, and unique in proscribing modern health care for adherents, although there was considerable variability among different Apostolic sects. We also found that the Johane Marange Apostolic Sect consistently communicated these views while other Apostolic sects tended to either be more mixed on their position or more like non-Apostolic churches, which do not forbid and often encourage HIV testing, child immunizations, and modern family planning methods for married couples. Surprisingly, even in the Marange sect, one young woman reported maintaining church membership while openly visiting clinics for care. This corroborates another recent report suggesting that even some ultraconservative Apostolic sects may be softening positions harmful to the health of women and children (Nyamanhindi, 2013).

Some pressure for change appears to be coming from women within the sects. For example, all Apostolic participants believed it was important for married couples to know their HIV status even though about a third said their churches forbade them to go to clinics. Since women from other churches are more likely to cross over to Apostolic sects than vice versa, perhaps they are bringing more liberal attitudes along with them. It should be noted, however, that the Johane Marange woman who defied her church’s ban on western clinics was a life-long member.

On the other hand, the fact that more women enter than leave the Apostolic sects also suggests that something about these faith communities is attractive. In a previous focus group study of one of the semi-conservative Apostolic Sects, we found that congregant women greatly admired the woman elder as a self-sacrificing wife and mother who also helped and cared for others (Mpofu et al., 2011). They noted that people joined the church when they had difficult physical or spiritual problems that were not helped by western medicine but instead were relieved by spiritual healing. Although all aspects of their lives were subject to strict female role expectations, membership also engendered a sense of belonging, purpose, and community.

Likewise, a study in nearby Mozambique that used a social capital framework to examine church communication about HIV found that Spirit-led churches were more likely to have strong bonding ties, gluing members together, while mainline Protestant churches were more likely to have weak, bridging ties that fostered connection with outside groups (Agadjanian & Menjivar, 2008). Mainline churches were described as more ‘worldly’, both in terms of their ideological flexibility as well as their political connections with the secular establishment, and having greater social heterogeneity compared to Spirit-Led Churches, which were described as more insular. The insularity of Zimbabwe ultraconservative Apostolic sects, however, may leave some young women in very difficult situations, and at the discretion of their husbands in seeking health options for themselves and their children, unless they are particularly assertive and in a more tolerant church.

A limitation of our study is the small sample sizes after categorization by Apostolic vs. non-Apostolic and by specific denominations. However, modest sample size is often inherent in qualitative research, when greater depth of exploration is needed because the experience of groups – particularly hidden groups, such as orphaned young women in ultraconservative Apostolic sects – is largely unknown. Our study design did not permit comparisons between young women who were orphaned in childhood and young women in general. However, orphans are a large and particularly vulnerable group in Zimbabwe and merit-specific attention. Our study was limited to interviews with young women as we were interested in their perceptions about how their religious affiliation might affect their marriage choices and reproductive health behaviors. We did not confirm these perceptions by interviewing church leaders about denominational doctrine or their own attitudes toward marriage and reproductive health practices. Also, findings cannot be generalized beyond orphaned Shona young women in eastern Zimbabwe. Nevertheless, our findings are consistent with previous, unpublished reports about Zimbabwe Apostolic vs. non-Apostolic sects (CCORE, 2011; Nhamo et al., 2011).

Despite the limitations, the study provides a rare and much-needed window into marriage and reproductive health practices for young women in rural Zimbabwe. We found that participants in all religious groups – even those in more restrictive Apostolic sects – want access to modern health care for themselves and their children, and that some have taken matters into their own hands. In particular, our sample of young women universally endorsed the importance of HIV testing, even if they had never been tested because of religious proscriptions. Since all had been orphaned in childhood in a country with a severe and protracted HIV epidemic, they were uniquely vulnerable to HIV infection, and proscriptions against clinic attendance presents a major barrier to accessing HIV testing and treatment. Many Apostolic congregations, however, appear to be softening restrictions and offering more discretion to women in their options for reproductive health care. Recent reports suggest that even some ultraconservative sects have made commitments to improve maternal and child health by allowing their members to attend clinics and go to hospitals (Nyamanhindi, 2013). More research is needed to monitor changes in attitudes and practices among Apostolic denominations, and the effects on HIV outcomes and maternal and child health indicators.

## Note

1. We use the definition of religion from the African Religious Health Assets Program: a wide variety of comprehensive systems of sacred beliefs and practices, usually (but not always) issuing in religious institutions, groups, or organizations that range from fluid to codified, popular to formal, centralized to decentralized, communal to institutional (ARHAP, ‘Appreciating Assets: The Contribution of Religion to Universal Access in Africa’, Report for the World Health Organization, Cape Town, October 2006). http://repository.berkleycenter.georgetown.edu/061000ARHAPAppreciatingAssets.pdf. For this paper, we focus only on Christian religious churches and congregations, since our participant sample affiliated exclusively with such groups.
